# Proceedings Veterinary Medical Societies

**Published:** 1886-07

**Authors:** 


### Proceedings of Veterinary Medical Societies.

At a meeting of the Medico-Legal Society, held in New York City, Thursday, June 10th, presided over by the Hon. Amos G. Hull, Vice-President, in the absence of the President, Dr. Frank H. Hamilton, the following discussion took place after the reading of Dr. Spitzka's paper on hydrophobia.

Dr. Dulles expressed the pleasure it gave him to be present at the meeting to hear the interesting and instructive paper of Dr. Spitzka, and to see the animals upon which he had experimented. He thought these experiments were of peculiar value as filling up a gap in the argument of those who did not believe in the specific nature of hydrophobia, nor in the theory of preventive inoculations with a so-called virus, as done by Pasteur. It showed that by the inoculation of matter which had no "specific character," symptoms could be produced such as Pasteur relies upon as evidence of rabies. He was opposed to the publicity given to the claims of Pasteur, and to the credulity with which they have been accepted. He believed that the more hydrophobia is talked about, the more prevalent it is. One of the effects of Pasteur's work has been an enormous increase in the number of cases of persons who considered themselves to have been inoculated with the virus of rabies, and to be exposed to the horrors of hydrophobia. If we admit Pasteur's claim, that he has saved more than nine hundred persons from hydrophobia in the last nine months, it appears that except for Pasteur the death from this disorder in France, would have risen from about fifteen a year to about four thousand! Pasteur claims to have proof that all of these persons were bitten by actually rabid animals, and that he has "cured" them of hydrophobia, in the stage of incubation. But those who do not believe in his theory, cannot admit any such claims. If we judge by what we know best about, we cannot have much faith in the madness of the dogs which bit Pasteur's patients. The Newark children still figure in the list of Pasteur's cures. We now know very well that the dog which bit them was not mad. There is not a particle of good evidence that it was. Of the bitten children, six in number, four went to Paris to be cured by Pasteur, and two remained at home and are just as well to-day as those who made the journey to Paris. Of the dogs bitten by this dog, which is said to have been mad, not one has since shown signs of rabies. Dr. Anna Kingsford, of England, has interested herself in this matter, and has investigated the cases of cure said to have come from her country, and she has not found one which will bear siftin g She calls attention to the point that when the cases which we can find out about turn out so unfortunately for Pasteur's credit, it is hardly worth while to trouble ourselves about those in regard to which we cannot get accurate information. It is a remarkable fact that Pasteur is constantly claiming infallability for his methods. In his last communication to a correspondent of the Boston Medical and Surgical Journal, he speaks in the customary positive way. Dr. Dulles had examined the subject of Pasteur's experiments, and found, that to justify his claims, he must have performed *without a single failure*, a number of series of experiments, each series requiring from 130 to 140 rabbits, and covering a period of nearly three years. Pasteur would have us believe that he has done all this without such failures, one of which would have broken up a whole series. But when we investigate the case upon which the success of his method hung—the case of the boy Meister, which gave him "cruel inquietudes"—we find that by Pasteur's own admission, one half of the material on which he relied to save the boy's life, and his own reputation, contained no virus at all! It is a curious illustration of Pasteur's carelessness, that, in speaking of the eleven inoculations which he made on this boy, he says that five of them, on being tested subsequently, were found to contain virus, five to contain none, while he omits altogether to account for the eleventh. And it illustrates Pasteur's forgetfulness, that in spite of this record, he even yet speaks as if his method were infallable. Is it remarkable that one should doubt this, in view of the fact that it disappointed Pasteur in half of the crucial tests in the case of the boy Meister?

Dr. Dulles said he had elsewhere gone in detail into various questions in regard to hydrophobia, and he wished to emphasize the point that evidence must be estimated according to its quality, and not its quantity. He believed that there was no more or better evidence, to support the specific theory of hydrophobia now, than there was a hundred years ago to support the belief in witchcraft. There were, in those days, a large number of respectable and intelligent witnesses, to prove the existence and the dangerous effects of the "evil eye." There were people who would die in the faith of having seen witches riding through the air. Such opinions are discarded now, and he believed that in fifty years the specific theory of hydrophobia would be abandoned. Certainly, if thinking men set their faces against the false notions in regard to it, which are too much proclaimed at the present time, the number of cases of hydrophobia would be much lessened. The number at present was very largely made up of three kinds of cases: those in which the spmptoms were due to some distinct and recognizable disease, those in which they were due to some form of mania, and those in which there was a pure state of fright. If these are well understood, and all cases attributable to them are eliminated from the discussion, as they ought to be, then there may be left a small residue, though he hoped there would be none, in which the symptoms would have to be explained in some other way.

Dr. I. N. Quinby said: *Mr. President and Gentlemen:* The call from your presiding officer for me to say a few words on the profound and varied questions involved in rabies, which important question is now agitating the community, comes to me very unexpectedly; for I came here to listen rather than to debate. But I will venture to say, however, that I am fully in accord with the sentiments and acts brought forward by Dr. Spitzka,—in his very able and important paper, and with his laudable efforts to let some true light, which is so much needed just now, on this occult and much misunderstood question of hydrophobia.

When we consider fully the influence of fear, anxiety, fright and joy upon the nervous centers; when we consider that persons have died within a few hours from fright; when we know that persons in robust health, may be transformed, by the unexpected loss of friends or possessions into permanent loss of health, into convulsions, loss of reason, even to committing suicide, etc.; may this not help us to explain why in times of Hydrophobic scare we have many cases of spurious hydrophobia (Lyssaphobia)?

No Physician however profound, no Neurologist, whatever may be his experience, has been able yet to fathom the mysterious workings of the nervous system under the profound agitation of the mind, or to discover what influence this agitated mind may have, through the reflexation of the nervous system on morbific agents that may have already been planted in some of the organs of the body. I am fully convinced that many of the reported deaths from hydrophobia (Lyssaphobia) have been brought about through fear and fright.

I fully concur with the sentiments expressed in Dr. Spitzka's paper in reference to the unreliability of the reported cases of cure by M. Pasteur, as in most of the cases sent to him it was not proven that the patient had been bitten by a rabid dog. Certainly there was no reliable evidence that the children sent from Newark, N. J., were bitten by a rabid dog, neither was there any evidence that Miss Morosini was bitten by a rabid dog, and the *post-mortem* evidence, revealed by A. F. Liautard, M. D. V. S., were of no practical value as bearing upon hydrophobia.

I have had some experience in the examination of dogs said to have died from hydrophobia. I will detail the result of the *post-mortem* of one which, with some modification, will be the history of all: My little son had a Spitz dog, the character of which, without any apparent cause, was suddenly changed from a playful to an irritable snappish mood, refusing either to eat or drink, frothing at the mouth, and snapping at everything that came in its way, and finally on the second or third day went into violent convulsions. While in these convulsions I put it under the influence of chloroform, keeping it under the influence for half-an-hour at a time; but after coming out of the influence of the anæsthetic it would go off into convulsions again, and continued in that condition for three or four hours, when death put an end to its sufferings. It was said by all of my family and the neighboring physicians that this was a genuine case of hydrophobia. But, being skeptical myself upon the subject I made a *post-mortem* and discovered that a *sharp chicken bone about one and a-half inches long* had *perforated* the *intestines.* In addition to this chicken bone which was the prime cause of this dog's death, with *characteristic* symptoms of hydrophobia, there were other marked intestinal lesions with as much of the *characteristic evidence* of *hydrophobia* as that discovered by Dr. Liautard, viz., congestion of the fauces, empty and congested condition of the intestinal tract, stomach containing straw, worsted from carpet, &c., &c. ; kidneys congested, bladder inflamed, contracted, and empty, effusion in brain and along spinal cord.

This hydrophobia scare subsided, not as rapidly, however, as it arose.

This is a fair sample, I think, of the kind of hydrophobia that nine of the canine species are afflicted with. One great good, Mr. President, I think will result from Dr. Spitzka's valuable paper, namely—that it will call the attention of the profession and the public sharply to the fact that many of the persons bitten by sudo-hydrophobic dogs will not have hydrophobia because the dogs were not rabid, and also to the importance of preserving the dog that has bitten a person long enough to know whether or not it develops true rabies, or, better still, until the dog dies of the disease, which would settle all doubt as to its true nature.

The action of Dr. L. D. Bulkley and Dr. Liautard smacked rather more of a sensation than of a scientific procedure, and would tend to do more harm to the community than good to the patient. *What evidence have we that the virus used* by M. Pasteur is the *true virus of rabies?*

The British Hydrophobia Commission, which is now investigating the question, are still in doubt in reference to this vital point, and however valuable the labors of M. Pasteur be, yet until this point is settled, that part of his investigation will be clouded with doubt and uncertainty.

Dr. M. G. Dadirrian said: I am from Constantinople, and have practiced medicine there, and have traveled in Asia Minor. I saw more dogs in the streets of Constantinople, than there are doctors in this room, If any of you gentlemen have ever been in that city, you will have seen as many dogs, at once, on the street, as there are trucks on lower Broadway. These dogs are born, live and die in the streets. They are very thin, and dirty and emaciated, but they do not have hydrophobia, though they quarrel and fight with each other, both day and night, over a small piece of bone or food. They are supported by small pieces of bread that the religious people think they ought to give them. During my practice in Asia Minor, I did not see any cases of hydrophobia.

A number of doctors used to meet together in Constantinople and talk about hydrophobia. Among this number were German, French, English and American doctors, I have never heard from any of them that he had seen a case of hydrophobia, though that was in a country infested with dogs. Very many of the people there, have the idea that there is such a thing as hydrophobia. The people speak of it as "Mad Dog." I think it is more imaginary than anything else. Now, to show that we would know of the existence of hydrophobia, if it existed at all, I will tell an instance. The Turks don't believe in the doctors, but when they get sick, and think they are going to die, they send for them. So, if there was any hydrophobia some of us would see it.

I read in the Constantinople papers, some time ago, that there had been a good deal of talk about hydrophobia, but I did not read an account of anybody dying.

The Turkish Priests will not permit the destruction of these dogs, or the taking of them from the streets. They claim that it is necessary to keep the dogs in the streets, because their religion tells them that when a man sins against God, and goes and asks pardon the way he knows his sins are forgiven is when he goes to a bakery and buys two or three loaves of bread and offers them to the dogs to eat, if the dogs eat the bread, his sins are forgiven. The people also provide tubs for the dogs to drink from, so that they will not become mad. Sometimes the people are bitten by the dogs; in such cases the people keep the dogs and do not kill them. This being so, we had come to the conclusion, before we heard anything of Pasteur, that we hardly believed in Constantinople, that there was such a thing as hydrophobia.

Dr. W. E. Brill said: As one who has paid some attention to the subject of hydrophobia, to the question of its existence in man, and one who is deeply interested in the discovery of

Pasteur, I am pleased to have listened to the paper of this evening, which expresses my views of the subject in every particular. With Dr. Spitzka and a very few others, I am on the minority side of a question which has been received by almost the entire world as truth. I am fully aware that our position is not very enviable, and that like all dissenters, we expose ourselves to the ridicule of the rest of mankind.

I would be the last one in the world to disparage the work done by Pasteur in other fields, work well done, and with good results; but to accept a theory as truth, simply because it comes from a man who has demonstrated some other scientific truths, is not only dangerous, but unreasonable, and I don't intend to be in the fashion of the present, which follows this method.

You may say: why surely he has proven his discovery by the fact of so many successful inoculations. But irrespective of my belief that there is no hydrophobia-producing virus residing in any tissue or secretion of a rabid animal bitten, but only in the imagination, let me argue the above on the same statistical ground. First, of fifty-nine animals inoculated by Hertwig, only fourteen were infected, three or four inoculations being necessary to secure infection in some cases, showing thus the immunity from the poison, and the difficulty of producing its effect on the animal to be very great. Second, in the year 1881, Gastier * made numerous experiments and arrived at the conclusion that the contagious element in hydrophobia was contained in the saliva of the dog, and that inoculation with the parotid or sub-maxillary gland, the brain juice, the stomach contents, the muscles and the pancreas led to no infection. He cultivated the virus of a hydrophobic dog in normal saliva, and vaccinated guinea pigs with it. These then died in from four to twelve days, but on vaccinating back from the saliva of the deceased guinea-pigs to dogs, he was *unable* to produce hydrophobia; only an ordinary septic influence resulted.

We are all aware that the character of the secretion is altered in an animal enraged or in fury. The accidental bite of a human being is not septic, but I have seen some of the worst forms of lymphangitis and cellulitis occurring in persons

---

* *Journal de Medecine Veterinire de Lyon*, 1881, *p.* 168.

injured by a bite during a fistic encounter, but never a nervous disease allied to hydrophobia. The infection from certain snake bites is often attended by fatal results, but never by similar phenomena attributed to a bite from a rabid dog; and the bite from other animals do not present any other features than those just mentioned; then why should that of the dog manifest a specific result in the form of a special grave nervous affection?

Even in what authorities regard as real hydrophobia in man it is remarkable that a large group of *symptoms* are of a kind *not* found in the animal from which the tissue is transmitted, nor, indeed, in any other animal subject to inoculation. It is also remarkable that these symptoms should happen to be the *traditional* ones of dread of water, spitting out saliva and terror. I quote the following extract from an Austrian treatise, which has had the honor of an American translation.*

"This feeling of abhorrence of water may increase to such a degree that a paroxysm will develop from a mere sight of water, from contact with it, from hearing the noise made by pouring it from one vessel to another, or, perhaps, from merely hearing compresses wrung out. * * * If the patient attempt to drink, or if the skin be moistened with water, spasms develop, which are attended with asphyxia, convulsions of the pharynx face and entire body, and a condition of intense excitement."

Of 216 cases of hydrophobia in man collected by Thamayn, six terminated in recovery; in other words, one out of thirty-six cases of what the authorities would regard as undoubted transmissions, got well without being "Pasteurized." I believe the results of "Pasteurization," if that procedure be shown to be entirely harmless (I myself should fear septic infection by his method) are no better than those due to nature. Pasteur includes among his alleged cures such cases as the Newark children and the historical one of Joseph Meister, who, it is now definitely established, had no hydrophobia at all, and had been bitten by dogs inferentially free from that disease.

Almost every day brings us news of a death of a patient under Pasteur's treatment. One of the Russians inoculated

---

* "A Clinical Treatise on the Diseases of the Nervous System." By M. Rosenthal. Wm. Wood & Co., 1879. Vol. ii., p. 100.

by Pasteur and discharged from his laboratory as secured against the disease, died of rabies on the journey home. This makes up a total of twenty-six persons bitten by mad wolves, and treated by Pasteur's method within the time prescribed by him, in all five deaths, or nearly 25 per cent. This percentage is as large as has ever been reported in cases bitten by dogs, I believe. It is not surprising that M. Pasteur wishes to exclude the persons bitten by wolves from his statistics, and rests on the 907 cases (April 29, 1886) which, like the boy Meister or the Newark children, had not been bitten by mad dogs at all. It is difficult, in view of the medium through which Pasteur's statements reach us, to learn what his exact views are ; but, if it really be true that he has stated, as was reported by the cable dispatches, his method to be efficacious against the dog rabies and inefficacious against the virus of the wolf, I do not think that I could present to you a more inconsistent statement to prove my previous assertion, or that we would be justified in discussing his method at all.

When Pasteur first promulgated his discovery, he guaranteed to cure any and all cases of hydrophobia that might occur, without reference to the time elapsed after the reception of the bite. When he met with the first death under his treatment, he stated that the patient arrived too late, that the virus had already produced the disease, and so with occurring reverses, he finally settled, after fixing the time at thirty-six days, upon the period of fifteen days as the incubatory stage. The daily press of June 7th reports the case of a death of a farmer from Roumania while under treatment by M. Pasteur. The Roumanian was bitten by a rabid dog on May 11th, and reached Pasteur on May 25th. After eleven days' treatment he showed symptoms of hydrophobia (June 5th), and died after forty-eight hours. The dispatch says, "This man's death and all the circumstances attending his case are held to upset the theory about the period required for hydrophobia to incubate, for the farmer was under M. Pasteur's treatment a long while before the expiration of the time heretofore deemed requisite for the poisonous saliva to obtain control of the victim's system. Dulles,* who has shown the fallacious argumentation and un-

* "Comments on Pasteur's Methods of Treating Hydrophobia." By C. W. Dulles, M.D. The *Medical Record*, Feb. 13, 1886.

trustworthy character of Pasteur's experiments, says in this connection, " how soon the short space of fifteen days may be reduced to fifteen minutes, we may tremble to contemplate, in view of the rapid rate of reduction which has prevailed thus far."

In view of this remark, I would hence say Pasteur institutes would have to be situated not only in every capital city, but in every village, nay, in every house and at every cross-road, if the persons bitten were to be inoculated in time.

Lorinser,* Dulles,† and other critical reviewers of Pasteur, have stigmatized his methods as imperfect and his manner of argumentation as faulty. I believe that no one will venture to deny that the rapidity and confidence with which he proclaims successive new discoveries tends to create the suspicion that they are not always properly matured.

I am aware that the objection that Pasteur is himself not a physician, aside from the fact that a medical degree is not a *conditio sine qua non* of biological ability, can be met by the fact that the first two boys inoculated by Pasteur were examined by no less a one than Vulpian, and pronounced to be inevitably doomed to die of hydrophobia unless Pasteur could prevent it. But this very assertion of Pasteur's friends shows how absurd it is to allow ourselves to be biased by the weight of a great name. If a medical student had made the statement of Vulpian and Grancher on the same ground that they did, namely, because there was severe laceration and many bites, it would have been justly stigmatized as puerile. There is no way of telling that a person is going to become hydrophobic before the outbreak of the disease.

If Pasteur is pursuing his present inquiries in a philosophical frame of mind, conscious of a great discovery, which is sure to be recognized, as all truths are, does the language which he recently used indicate it? The correspondent of the Boston *Medical and Surgical Journal* reports it as follows : " In reply to my remark that the drift of opinion in America seemed to be that it is necessary to wait for proofs of protection in

---

* *Wiener Medizinische Wochenschrift*, 1885, No. 51, p. 1562.

† "Comments on Pasteur's Methods of Treating Hydrophobia. *Medical Record*, Feb. 13, 1886.

those already inoculated, he (Pasteur) said with great energy, 'Wait! I have no objection. I am waiting. But consider this; in October last I began systematically to inoculate human beings. Up to this moment (April 29th) we have inoculated 907 persons. How many deaths? One. One only (for the Russians cannot justly be counted), and when this one, the Pelletier child, came to me, it was too late. Before I began to inoculate human beings, how many of those bitten by mad dogs in France died? Sixteen per cent. Since last October, only one death in 907 cases. What more can you ask? Scientific men once doubted my having discovered a means of preventing cholera in chickens, and charbon in sheep. When I announced my discovery at the International Medical Congress in London, Koch said "pooh," and Chauveau simply loaded me with sceptical objections. Do they do it now? Let the fact that 500,000 sheep are inoculated every year in France for peasants who fight to save a two sous piece, and yet must pay four sous per head for inoculation every spring, be the answer. The Germans are angry, especially Koch, because France has the merit of this new discovery of protection against rabies. They think because they conquered our country in 1872 that they can be our masters in this field, and therefore they spread broadcast their scepticism against me. They lack generosity. They are behaving like children.'"

Whoever heard of the Germans objecting to the originality of Voltaire, De la Mettrie, Laplace, Arago, Bonpland, Cuvier, St. Hilaire, Cruveilhier, or Charcot, because a former monarch of the French had ravaged the finest province of Germany with such unprecedented barbarity that from the Pope to the Highlanders all Europeans stood aghast.

But questionable as his statistics are, can he show better results than Youatt, whom Hammond quotes,* as employing cauterization in over four hundred persons bitten by rabid animals, and never unsuccessfully. He employed it four times on himself, but finally committed suicide under fear that he was going to have the disease.

It is fortunate to the cause of scientific truth, that the warmest admirers of Pasteur should be among the laity, and not

---

* A Treatise on the Diseases of the Nervous System, 6th edition, 1876.

among medical men, but he is also very unfortunate in his medical adherents, for it is these admirers, whether with or without his sanction, have resorted to advertisement, which is in no sense different from that of a circus show. Whenever public interest threatens to flag for a moment, the cable brings us news of some new order of merit by the Emperor of Brazil, or a new grant to the Pasteur institute, or the name of some distinguished physician who goes to Paris to study Pasteur's methods. It is exceedingly unfortunate that some of these grandiloquent announcements were entirely false, while others were misrepresentations. Thus it was stated that Duke Carl, of Bavaria, together with his Duchess, both of whom are practitioners, were about to proceed to Paris to study Pasteur's system. The former's assistant, Dr. F. Tausch, was forced to declare that this was without foundation.* It was also reported that the Austrian government had sent a delegate to Paris for the same purpose. In truth, it was a private person, a Princess Metternich, whose record is about as savory as that of ex-Queen Isabella, of Spain, and who is ready to play any part from a ballet girl, to a bazar-patroness in the interests of charity to others and notoriety for herself, who sent Dr. Fritsch on this errand.

It is remarkable, considering the enthusiasm reigning among the adherents of Pasteur, that there should be so much jealousy and distrust among them. It is particularly the American disciples who show a feverish ambition for leadership, and an impatience of rivalry, which, considering the slight amount of expenditure of intellect involved in cataloguing dogs and rabbits, and making hypodermic injections, seems to indicate a very unhappy frame of mind.

In conclusion, I cannot do better than to again have recourse to Dr. Dulles'† most admirable review of the methods of Pasteur, who says: "I venture to express my opinion that Pasteur's so-called method of treating hydrophobia, before the outbreak, *does* appear to be founded upon untrustworthy experiments and unsound reasoning, and ought to be rejected and condemned, in the interests of humanity as well as of science.

* British Medical Journal. May 22d, 1886, p. 991.

† Opus. cit.

. . . . . . . Pasteur's arrogance, impatience of correction, ignorance or disparagement of what others have done, his secret methods, his hasty assumptions, his illogical conclusions —have made him many scientific and personal enemies. But, after all, Pasteur is the man who placed on a new and apparently secure basis our knowledge of the nature of ferments; who disproved the theory of spontaneous generation; who laid the foundation upon which rests the whole system of antiseptic surgery, for the establishment of which the name of Lister will be justly immortal. Such a man is Pasteur, and, whatever of error may seem to mar some of his last endeavors, and however the future may adjudicate some of their claims, he seems to me to deserve all the honor he has received for his unwearied labors, and his magnificent achievements in the cause of science."

"His very achievements in the right direction, however, make any error on his part all the more dangerous to the cause of truth, and makes it all the more the duty of thinking men to sift the evidence upon which he rests his extravagant claim of having discovered a means of preventing the outbreak of hydrophobia."

Dr. H. W. Biggs said: The sentiment to-night has been against the existence of hydrophobia, and entirely against the work of M. Pasteur. I wish to make some statements in correction of this, and to express my entire disapproval of the conclusions reached. In the Newark case, six children were bitten, and four were sent to Pasteur and returned cured. In another case, a lady was bitten by a dog in the street, and the idea of hydrophobia never occurred to her at all. Some weeks after, symptoms of hydrophobia manifested themselves and she died. It is said that the diagnosis of that dog rested entirely on the authority. The diagnosis did not rest upon the autopsy of the dog. It is admitted that the four children that went to Paris have not died, neither have the two that remained. Now that the latter two children lived does not prove that the other four did not have the hydrophobia.

Another case, is that of the keeper of the dog pound. He was bitten. I believed at the time that it was a case of hydrophobia. I agreed with the diagnosis that it was a case of hydrophobia. I made the autopsy the following day. I made inoculations from this dogs virus on other dogs and on rabbits. One of my dogs, so inoculated, had signs of paralysis nineteen days after. I did not believe it was a case of rabies, for it developed after the period when rabies could have been expected. I have made other inoculations and have had no better results. Of course, there was foreign matter found in their stomachs, but that is to be expected in all dogs. The other dogs inoculated are doing well. Dr. Law also made some inoculations, with about equal results. One of the animals died with paralytic symptoms, of course, this would go to prove that the dog did not have the rabies. I finding the presumption to be against a case of rabies, concluded that it was not, and thought that we had made an error in the diagnosis. Dr. Spitzka has taken the stand, in his lecture, that they are the symptoms of rabies in dogs, as they occur after inoculation. I think they are not the proper symptoms, and certainly, are not those demonstrated in the Pasteur laboratory.

As regards conclusions and opinions of Pasteur and his work, I was in Pasteur's laboratory for two weeks, and watched his method. I think the question about hydrophobia is yet to be determined, though the evidence, to my mind, is strong and conclusive. His results, so far as I was able to determine, were exceedingly strong. I think he has proven the existence of hydrophobia or rabies in dogs. At all events he has produced some disease not at all like septicæmia, which has a regular incubation, and a peculiar symptomatology.

Dr. Spitzka being invited to close the discussion, did so with the following remarks. I feel indebted to all who have spoken; the majority have not only endorsed my conclusions, but gone much further than the paper in condemning what I venture to regard as a delusion. I am particularly obliged to Dr. Biggs for his candid admission of many facts, which, had I known of them before, I should have incorporated in the paper. All the more do I regret that Dr. Biggs should have discussed the paper under the impression which he seems to have labored under that he was in opposition to its conclusions. It is true that the discussion has taken a direction against the existence of hydrophobia, and against the correctness of Pasteur's views. I cannot regret this, as my private bias of opinion is against the latter. But as I have not ventured to discuss this method in the paper, I claim immunity to criticism. I do not think Dr. Biggs heard the earlier part of my paper, or he could scarcely have gotten the impression that I claimed these dogs to represent true cases of hydrophobia. I so distinctly repudiated the existence of true hydrophobia here, and accentuated the absurdity of Liautard's claim to that effect so strongly that I am surprised at the misconception. I claimed nothing more than to have imitated Liautard's—if you will—*bogus* hydrophobia! That I have most certainly done, and I am pleased to find Dr. Biggs agreeing with me that these symptoms of Dr. Liautard's dog were those of ordinary brain disease and not of hydrophobia. It was to prove this very proposition I read this paper and made these experiments.

As regards the Newark epidemic I must dissent from some of Dr. Bigg's inferences. He speaks of two cases of fatal hydrophobia in residents of Newark, antedating the "scare." In the absence of reliable medical records of these cases, I cannot regard them as anything else than ancient history. To strengthen Pasteur's claims to having cured the Newark children, he says that the two who did not go to Paris were not so severely lacerated by the teeth of the dogs as the others. I believe the general professional opinion to be that wounds bleeding freely are less likely to prove poisoned wounds than slight punctures or scratches which do not bleed freely, and on this score, Dr. Biggs' inference as to the relative immunity of those who remained behind cannot be sustained.

His statements about George Neall, the Newark pound-keeper are exceedinglyly valuable. If we assume Pasteur to be correct, that inoculation of hydrophobic brain and cord will produce hydrophobia in animals inoculated according to this method, then the negative results of Drs. Biggs and Law prove conclusively that George Neall at least had not hydrophobia. If he had not hydrophobia my position that a much larger number of cases of fatal hydrophobia exists than is usually imagined, is fortified by Dr. Biggs' very candid admission.

I cannot agree with Dr. Biggs in his stricture on Dr. Dulles figures. It is true that some of the 907 cases were not from France, but the majority were. His noteworthy conclusion, with a slight change, remains uncontroverted. I cannot censure Dr. Dulles for assuming that Pasteur ignored the fact that only 15-25 per cent. of persons bitten by rabid dogs go mad. Dr. Biggs says that Pasteur is aware of this. If so, there is no indication of it in his proclamations. He uses the expression "I have cured" to the reporter of the *Boston Journal.* He also says—to cover his failures—that the bite of the mad wolf is more fatal than that of the mad dog. To prove this, he picks out some cases running back as far as 1806 and 1788, showing that 100 per cent., or nearly that, of those bitten died. He seems to forget altogether the surgical nature of the wounds. A wolf makes an ugly job of the person he attacks. If he escapes with life he is probable remote from the little hamlet where he resides; hemorrhage is apt to be extensive; the bites are usually inflicted in cold weather, which has a bad effect on wounds, and medical aid is administered late, if at all. Under these circumstances exhaustion, blood-poisoning, erysipelas and tetanus ought not to surprise us.

With regard to Dr. Kretchmar's case, I stated it, to avoid argument, to be one of hydrophobia. Yet is it not strange that this should be the only case in a large city like Brooklyn? I do not think that terror and expectant attention can be excluded. The attack by the second dog so shortly before the fatal issue, was, at least, unfortunate. I will however say, that of all cases reported within the year, here, his is the one which best corresponds to the typical picture described in text books. Dr. Bigg's patient, if I am not mistaken, was regarded as a case of alcoholic delirium.

Dr. Biggs. I suggested that, but was not able to find grounds for the belief.

Dr. Spitzka. I suppose Dr. Biggs was in just such a position as I would be in to-morrow, or in which Dr. Kretschmar was, that of never having seen a true case before, and of being compelled to make the diagnosis from our reading knowledge? (assented to.) I must admit being puzzled by the question as to the existence of the real disease. Those who advocate it are certainly inconsistent in a high degree. The excellent authority whom I quoted: Law, admits that there are no differential criteria, between the real and the spurious affection. Do you know of any other contagious disease which can be so closely simulated? Years ago, when advocating the reality of lyssa in man, in a conversation, I urged its transmission to cattle, as proof that there was an element in it, independant of mental influences. To-day I cannot maintain that, in view of the singular statement reproduced in Law's essay, that the convulsive seizures in herbivora and omnivora, are provoked *by the sight of a dog!*

The fact has been advocated, too, that the discussion was rather one-sided. I must admit that to be true, and no little surprise to myself. I certainly took every measure to ensure the presence of the members of the Pasteur institute, but their leaders have excused their inability to attend on varions grounds. They were invited officially, as well as by myself, individually, and the notices of the meeting were abundant, and my intentions known. If I have shown, irrespective of the question of true hydrophobia, that a larger numb r of deaths than the dog is responsible for, are due to terror and expectant attention, and can be avoided by suppressing such inexcusable scares as were started at Riverdale and at New York, my purpose will have been accomplished.

A vote of thanks having been passed, a committee of three was appointed, on motion, by the chair, to further consider the matter and report.

During the reading of the paper, the four dogs, named A, B, C and D, were exhibited, the dog B, particularly showing paraparesis on being urged to jump off a chair, while dog C, showed stupor and manége movement, somewhat hampered by the confined space. The brains of dogs E and F, the latter of whom had died delirious that morning, were exhibited, showing purulent meningitis. Dog A was chloroformed to death by Dr. Hamill, and Veterinary Student Atcheson, and the brain exposed by Dr. Brill. The dura was found to have united in a milk-white linear cicatrix. There was an adhesion to the leptomeninges and a spot of inflammatory red softening, of the size of a three cent piece, deeply extending into the brain substance. A blood clot was found lying further forward, and the entire hemisphere, of the side of the operation (right) was injected and slightly tumefied.

